# Recursive partitioning models for linkage in COGA data

**DOI:** 10.1186/1471-2156-6-S1-S38

**Published:** 2005-12-30

**Authors:** Wei Xu, Chelsea Taylor, Justin Veenstra, Shelley B Bull, Mary Corey, Celia MT Greenwood

**Affiliations:** 1 Genetics and Genomic Biology, Hospital for Sick Children, Toronto, Ontario, Canada; 2 Population Health Sciences, Hospital for Sick Children, Toronto, Ontario, Canada; 3 Department of Public Health Sciences, University of Toronto, Toronto, Ontario Canada; 4 Samuel Lunenfeld Research Institute, Mount Sinai Hospital, Toronto, Ontario Canada

## Abstract

We have developed a recursive-partitioning (RP) algorithm for identifying phenotype and covariate groupings that interact with the evidence for linkage. This data-mining approach for detecting gene × environment interactions uses genotype and covariate data on affected relative pairs to find evidence for linkage heterogeneity across covariate-defined subgroups. We adapted a likelihood-ratio based test of linkage parameterized with relative risks to a recursive partitioning framework, including a cross-validation based deviance measurement for choosing optimal tree size and a bootstrap sampling procedure for choosing robust tree structure.

ALDX2 category 5 individuals were considered affected, categories 1 and 3 unaffected, and all others unknown. We sampled non-overlapping affected relative pairs from each family; therefore, we used 144 affected pairs in the RP model. Twenty pair-level covariates were defined from smoking status, maximum drinks, ethnicity, sex, and age at onset. Using the all-pairs score in GENEHUNTER, the nonparametric linkage tests showed no regions with suggestive linkage evidence. However, using the RP model, several suggestive regions were found on chromosomes 2, 4, 6, 14, and 20, with detection of associated covariates such as sex and age at onset.

## Background

Alcohol abuse and alcohol dependence are psychiatric disorders with severe physiological and psychological ramifications including liver disease, heart disease, gastrointestinal disease, depression, suicide, and homicide. In addition, fetal alcohol syndrome is a leading cause of mental retardation. A 1992 estimate put the economic burden of alcohol use in Canada at $7.5 billion, or 40.8% of the costs of all substance use combined [1]. Although relatively common (a 1992 study estimated alcohol dependence and abuse prevalence in the US to be 7%), the disorders are complex and the etiology is not well understood. Evidence for a genetic component to the disease stems from observations of familial clustering and twin and adoption studies. Many phenotypes associated with the risk of alcoholism, such as response to alcohol, maximum number of drinks in one sitting, and measurements such as brain electrophysiological measures are known to be related to underlying genetic factors and have been shown to cluster in families in which alcoholism is also observed. Furthermore, co-morbid states including depression, other substance abuse problems, and antisocial personality disorder have their own underlying genetic factors. Studying co-morbid states can facilitate the search for underlying genetic mutations by helping us to understand common etiologic pathways. Similarly, the development of new phenotypic measures such as behavioral responses and physiological reactions may further aid understanding of the phenotype-genotype relationship.

We analyzed genome-wide microsatellite data from the Collaborative Study on the Genetics of Alcoholism (COGA), supplied by the Genetic Analysis Workshop 14. There were 1,614 individuals in 143 pedigrees. Probands, recruited from chemical dependency centers, and their families were invited to participate in the COGA study. All of the participants were assessed on several domains, including alcohol dependence; other psychiatric disorders, such as depression and other medical illnesses; the participant's family history of alcoholism; and other behaviors. Diagnoses of alcohol dependence and other psychiatric disorders were established using a structured, comprehensive, diagnostic interview called the Semi-Structured Assessment for the Genetics of Alcoholism, which was developed specifically for the COGA study.

Most methods for nonparametric linkage (NPL) analysis require a fixed definition of affected status and can incorporate only a few covariates [2,3]. For any one susceptibility locus for a complex trait, it may be that the locus modifies risk through interaction with a covariate, or through a secondary phenotype or endophenotype that influences the primary diagnosis only indirectly. Here, we developed and implemented a method for simultaneously estimating linkage while choosing the covariates that are most tightly associated with the linkage measurement at that locus. This strategy may improve power to detect linkage and improve understanding of disease etiology.

## Methods

### Statistical model

In order to adapt the conceptual framework of a standard recursive partitioning (RP) (tree-based) model [4] for linkage analysis, we assess evidence for linkage with the affected-relative-pair model of Olson [3]. A likelihood ratio test statistic for linkage can be written as:



The likelihood ratio is summed over all the *n *informative affected relative pairs. The parameter *λ*_*i *_measures the excess risk to an individual who shares, at the marker locus, *i *alleles identical by descent (IBD) with an affected relative compared to the population risk [3]. *λ*_*1 *_corresponds to IBD = 1, *λ*_*2 *_corresponding to IBD = 2, and *λ*_*0 *_= 1. *f*_*ir*(*p*) _is the prior probability of sharing *i *alleles IBD for affected pair *p *of relative type *r*. For example, for sib pairs, the expected IBD sharing is (1/4, 1/2, 1/4) under the null hypothesis. g_*ip *_represents the estimated probabilities of sharing *i *alleles IBD based on marker data for pair *p*. The parameters *λ*_*i *_are estimated by optimizing the total likelihood ratio for all the affected relative pairs. This formulation unifies different types of relative pairs because expected allele sharing for any pair type can be expressed as functions of the same parameters *λ*_*i*_. This leads to a test of linkage deviation from the null hypothesis (no linkage) based on two parameters ***λ ***= (*λ*_*1*_, *λ*_*2*_) and 2 degrees of freedom.

For each pair-defined binary covariate *X*_*p *_(*X*_*p *_= 1 or 2), a likelihood ratio test of linkage in the presence of heterogeneity can be obtained by estimating two sets of parameters (). We therefore define a splitting rule, in the spirit of regression trees, based on identifying the covariate that gives the largest likelihood ratio test statistic for linkage with heterogeneity. This is implemented recursively until the subgroups are too small for further splitting. Again following standard RP model concepts, we used 10-fold cross-validation [5] to estimate the optimal tree size (total number of terminal nodes). The pairs were randomly divided into 10 equally sized subgroups; leaving out each subgroup in turn, the tree was grown on the remainder. The performance of the model can then be assessed in the 10% of the data that were omitted. Let ***λ***^***k***^_***t ***_represent the estimated relative risk parameters from cross-validation training set *k*, (*k *= 1, ..., 10), and covariate-defined subgroup *t *(*t *= 1, ..., *s*) with tree size *s*. For *s *= 1, there is only one set of *λ *estimates (corresponding to the root node of the tree), for *s *= 2 there are two sets, etc. Let *p *∈ *t*(*k*) denote the pairs in the *t*^th ^subgroup of the *k*^th ^cross-validation test set, where subgroups are defined by the tree grown on the *k*^th ^training data set. A measure of deviance can therefore be constructed, based on the testing data:



The optimal tree size is selected as the one with the largest deviance measure. The relative risk estimates used in the deviance calculation are those which optimized the likelihood ratio for splitting the tree in the *k*^th ^cross-validation training test set. After choosing the optimal tree size, we used a bootstrap algorithm to determine the consistency of particular covariate selections. When one covariate clearly defines linkage heterogeneity, most bootstrap datasets will select the same covariate. When several covariates are associated with the disease gene, bootstrap datasets may choose a variety of tree structures (configuration of a tree).

We calculated *p*-values for tests of linkage and heterogeneity assuming an asymptotic chi-squared distribution. The RP model provides tests of linkage with and without covariate-induced heterogeneity, as well as tests of covariate effects on the linkage. As currently implemented, this model places no plausibility constraints on the *λ *values. Hence deviation from the null hypothesis can show either excess allele sharing or decreased allele sharing.

### Application to the COGA data

Use of the COGA data set was approved by the Hospital for Sick Children Research Ethics Board. We used primarily the ALDX2 (DSM-IV) criteria to define affection status. We treated category 5 (affected) as affected; categories 1 (pure unaffected) and 3 (unaffected with some symptoms) as unaffected; categories 0 (unknown) and 2 (never drank) as missing. Based on this definition, there were a total of 726 informative affected relative pairs. In order to avoid working with highly dependent affected pairs within a pedigree, we sampled non-overlapping affected relative pairs from the same family. Therefore, we used 144 affected pairs in the RP model.

We defined 20 pair-level covariates using smoking status, maximum drinks, ethnicity, sex, and age at onset. We defined smokers as those with non-zero pack-years (smokers *N *= 914; non-smokers *N *= 467, missing *N *= 233). To differentiate between heavy and light smokers, we utilized a cut-point of 21.00 pack-years which represented the third quartile for all 1,381 individuals with available data. We then defined four pair-level covariates for smoking: 1) both smokers versus others, 2) both non-smoker versus others, 3) discordant smoking status versus others, and 4) both heavy smokers versus others. Note that "others" includes pairs with missing covariate information. A similar approach was taken to define binary covariates for other variables. Electrophysiological variables were not used in this analysis.

Multipoint NPL scores, the estimated IBD allele sharing, gip, and the null expected sharing for each affected relative pair, f_*ir*(*p*)_, were obtained from GENEHUNTER [6] using the microsatellite markers and the complete pedigrees. When families were too large, the default GENEHUNTER algorithm was used to drop individuals from the pedigree. We calculated the NPL scores using the "all pairs" score that summarizes sharing across family pair-wise relationships; in this score, the dependency between pairs is not a concern.

## Results and Discussion

**Figure 1:**
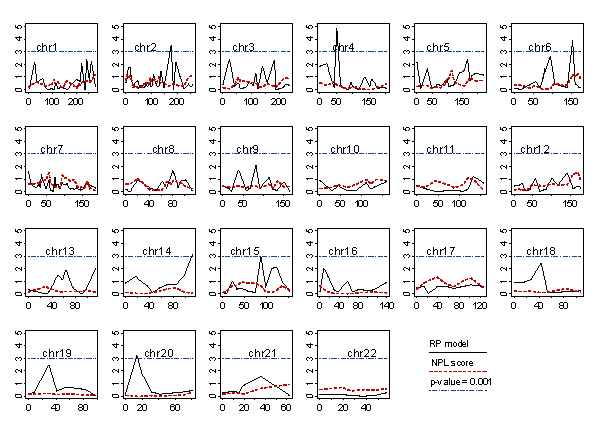
-log10(*p*-values) of NPL score and of the RP model.

**Figure 2:**
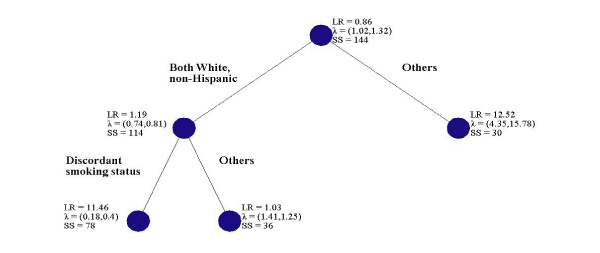
Final tree for D2S2275 (SS, sample size).

The NPL scores provided no linkage evidence (with criteria NPL = 3.1, *p*-value = 0.001; dashed lines in Figure 1 show -log_10 _of the *p*-values) [7]. We then applied the RP model on our selected non-overlapping pairs using the same microsatellite genotypes (Figure 1, solid lines). We found suggestive regions on chromosomes 2, 4, 6, 14, and 20 with *p*-values smaller than 0.001 (Table 1). There is good consistency across bootstrapped datasets for the choice of the first covariate. Figure 2 illustrates the final tree for marker D2S2275. Two subgroups show strong linkage/allele sharing: pairs where both are White but discordant for smoking status, and pairs where at least one member is not White.

Although NPL scores showed no linkage evidence on any of the 22 chromosomes despite a larger sample size (use all pairs), the RP data mining algorithm identified loci in regions that have been previously identified, which are on chromosomes 2 (D2S2275; 175.4 cM) [4], 4 (ABRB1; 51.4 cM) [8,9], and 6 (D6S495; 153.8 cM) [10]. The relative risk parameters measure marker-specific (i.e., locus-specific) increases in disease risk to relatives with particular IBD relationships. The estimates of relative risk make it possible to do some interpretation of the linkage evidence in subgroups; however we found that the chosen splits usually divided the sample into one group with excess sharing and a second with *λ *estimates that violated the possible triangle constraints. Interpretation of the results can be difficult, especially when pairs in the subgroups are concordant for their covariate values. We are planning to implement constraints on the allele sharing parameters.

The definition of "affected" is crucial for any linkage study. Expected patterns of allele sharing in linked regions vary with changes to these definitions. Our algorithm focuses on sharing between affected relative pairs, and hence, although we can find heterogeneity in linkage evidence, it is always predicated on the initial definition of affected status. It may be possible to construct better definitions of alcoholism from a combination of phenotypes.

Our algorithm as currently implemented assumes independence of relative pairs, but this is violated when multiple pairs are constructed from the same pedigree. To reduce dependency, we selected non-overlapping pairs, but this excluded a large number of relative pairs. Therefore, we could expect the NPL scores based on the full pedigrees to have better power. However, the NPL method found no linked regions, whereas our approach identified several regions also identified by others. In the future, we plan to develop appropriate methods for dependent pairs.

Despite the cross validation, any data mining algorithm is likely to find some false positive results. Therefore, additional strategies will be necessary to reduce false positive signals. For example, we might expect broader peaks to be associated with real linkage signals [11].

## Conclusion

We developed a recursive-partitioning model for linkage analysis to select covariates that are associated with the allele sharing in relative pairs. Cross-validation and bootstrapping are used to improve the properties of the model. In the COGA data, we were able to detect linkage signals involving covariate interactions that the NPL scores were unable to detect.

## Abbreviations

COGA: Collaborative Study on the Genetics of Alcoholism

∈: Is an element of

IBD: Identical by descent

NPL: Nonparametric linkage

RP: Recursive partitioning

## Authors' contributions

WX developed and implemented the RP algorithm under the supervision of CMTG. SBB and MC and drafted the manuscript. JV assisted with data manipulation and developed the plotting code. CT carried out the literature review under the supervision of MC. SBB and CMTG assisted in revising the manuscript.

## Figures and Tables

**Table 1:** Suggestive linkages region detected by RP model

Marker name (DECODE)	NPL -log10 (*p*-value)	Overall RP -log10 (*p*-value)	Splitting covariates (in split order)	Subgroup with largest likelihood ratio	*λ*_1 _and *λ*_2 _for this subgroup	Bootstrap proportion (first split)^a^
D2S2275 (Chr 2: 175.4 cM)	0.31	3.47	Ethnicity, Smoking	Not (both White non-Hispanic)	4.35, 15.79	14% (53%)
GABRB1 (Chr 4: 51.4 cM)	0.35	4.81	Sex, Age at onset	Both male; both early age at onset	0.20, 0.94	19% (59%)
D6S495 (Chr 6: 153.8 cM)	1.19	3.85	Smokers, Max drinks	One smokes, one not; one heavy drinker, one not	8.00, 5.40	19% (47%)
D14s51 (Chr 14: 111.0 cM)	0.043	3.14	Max drinks, Sex	One heavy drinker, one not	0.38, 0.01	35% (52%)
D20S50 (Chr 20: 13.6 cM)	0.003	3.20	Sex, Smoking	Both male; both smoker or both not smokers	0.29, 0.07	25%(54%)

^a^Proportion of bootstrap trees that selected the same covariates (proportion that selected the same covariate at the first split).
